# A Murine Model of Persistent Inflammation, Immune Suppression, and Catabolism Syndrome

**DOI:** 10.3390/ijms18081741

**Published:** 2017-08-10

**Authors:** Amanda M. Pugh, Nicholas J. Auteri, Holly S. Goetzman, Charles C. Caldwell, Vanessa Nomellini

**Affiliations:** 1 Division of Research, Department of Surgery, University of Cincinnati, Cincinnati, OH 45229, USA; pugham@ucmail.uc.edu (A.M.P.); njaute54@thomasmore.edu (N.J.A.); goetzmh@UCMAIL.UC.EDU (H.S.G.); 2 Division of Trauma, Critical Care, Acute Care Surgery, Department of Surgery, University of Cincinnati, Cincinnati, OH 45229, USA

**Keywords:** PICS, chronic critical illness, sepsis

## Abstract

Critically ill patients that survive sepsis can develop a Persistent Inflammation, Immunosuppression, and Catabolism Syndrome (PICS), which often leads to extended recovery periods and multiple complications. Here, we utilized a cecal ligation and puncture (CLP) method in mice with the goal of creating a model that concurrently displays all the characteristics of PICS. We observed that, after eight days, mice that survive the CLP develop persistent inflammation with significant myelopoiesis in the bone marrow and spleen. These mice also demonstrate ongoing immune suppression, as evidenced by the decreased total and naïve splenic CD4 and CD8 T cells with a concomitant increase in immature myeloid cells. The mice further display significant weight loss and decreased muscle mass, indicating a state of ongoing catabolism. When PICS mice are challenged with intranasal *Pseudomonas aeruginosa*, mortality is significantly elevated compared to sham mice. This mortality difference is associated with increased bacterial loads in the lung, as well as impaired neutrophil migration and neutrophil dysfunction in the PICS mice. Altogether, we have created a sepsis model that concurrently exhibits PICS characteristics. We postulate that this will help determine the mechanisms underlying PICS and identify potential therapeutic targets to improve outcomes for this patient population.

## 1. Introduction

Significant improvements in the diagnosis and management of sepsis or septic shock have decreased overall mortality rates [1]. While mortality is often utilized as a measure of overall outcomes from sepsis, it is imperative to recognize that individuals who survive sepsis do not necessarily experience a favorable recovery. Those that survive may progress to a state of chronic critical illness and suffer a prolonged hospitalization marked by multiple complications, poor wound healing, profound disability, and long-term neurocognitive deficits, often leading to the withdrawal of life supporting measures [2,3]. These patients frequently progress to a state that is commonly referred to as Persistent Inflammation, Immunosuppression, and Catabolism Syndrome (PICS) [4,5,6]. PICS can occur not only in patients who present with sepsis, but also those sustaining traumatic or burn injury. PICS is currently defined by prolonged hospital stays, the persistent elevation of pro-inflammatory cells [7], suppression of adaptive immunity [7,8,9,10], and ongoing catabolism as evidenced by significant weight loss despite adequate nutrition [4,11]. Currently, there are no therapeutic interventions for PICS, beyond supportive care. As such, there are no known interventions for PICS that occurs during the later stages of sepsis. Without a pre-clinical model, it is difficult to determine treatment strategies for these patients.

Murine sepsis models can recapitulate the persistent inflammation, immune suppression, and catabolism observed in humans. Yet, no animal models have exhibited PICS in their entirety and a large number only focus on the acute phase of sepsis. While PICS can occur after a wide variety of insults, we chose to first evaluate this process in response to sepsis. Once adequately modeled in animals, we will be able to validate these observations in humans. The current study tests the hypothesis that mice surviving a septic insult develop PICS similar to humans, rendering them more susceptible to secondary infections and inflammatory insults. Mice were evaluated eight days after CLP, which is a time point at which we determined that each component of the PICS syndrome is present. The successful completion of this hypothesis would be the development of a pre-clinical model amenable to testing therapeutic interventions.

## 2. Results

Here, we utilized a murine model of sepsis with a mortality of approximately 25–33% in each cohort (previously published) [12], which mimics the mortality rate in humans of 10–40%, depending on the severity of illness [1,13]. The majority of these deaths took place within 24–72 h of the cecal ligation and puncture (CLP) surgeries. The occasional septic cohorts that died outside this range were not utilized for this report. The following data are collected from mice that survived the initial insult.

### 2.1. Persistent Inflammation

**Figure 1:**
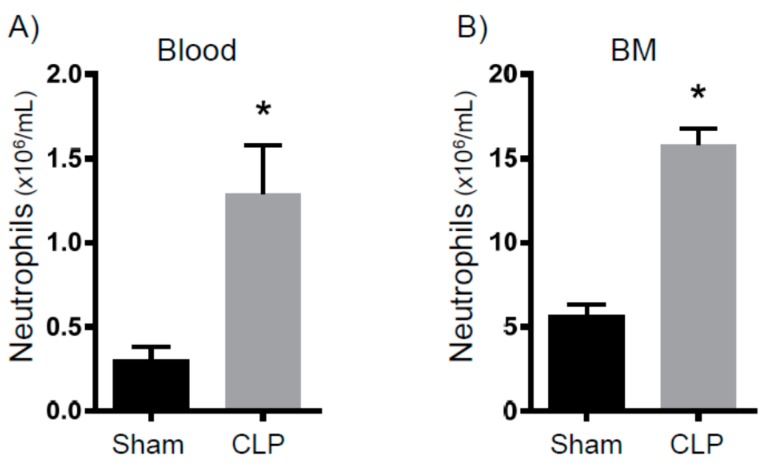
Leukocytosis and bone marrow myelopoiesis eight days after cecal ligation and puncture (CLP). Mice were subjected to either sham surgery (*N* = 13) or CLP (*N* = 17). Eight days later, animals were sacrificed and flow cytometry was used to characterize the number of (**A**) neutrophils in the blood and (**B**) neutrophils in the bone marrow. * *p* < 0.05 versus sham.

**Figure 2:**
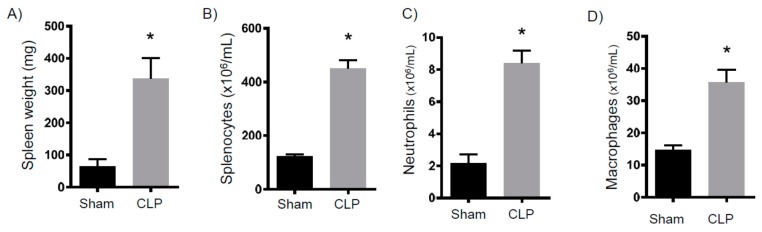
Splenic myelopoiesis eight days after CLP. Mice were subjected to either sham surgery (*N* = 14) or CLP (*N* = 20). Eight days later, animals were sacrificed and their spleens were analyzed for (**A**) weight, (**B**) total number of WBCs, (**C**) neutrophils, and (**D**) macrophages. * *p* < 0.05 versus sham.

To determine persistent inflammation, we analyzed myeloid cell numbers eight days after CLP. First, we measured the neutrophil numbers in circulation and within bone marrow (Figure 1). Compared to sham mice, we found a three- to four-fold increase in neutrophil concentrations. When next analyzing splenocytes, we observed that the spleens isolated from the CLP mice were strikingly larger and of an increased mass (Figure 2A). Additionally, these spleens contained more white blood cells (Figure 2B). Finally, spleens isolated from the septic mice demonstrated more neutrophils and macrophages (Figure 2C,D). Altogether, eight days after CLP, mice exhibited increased myelopoiesis as evidenced by increased myeloid cells in circulation, bone marrow, and lymph tissue.

### 2.2. Immune Suppression

**Figure 3:**
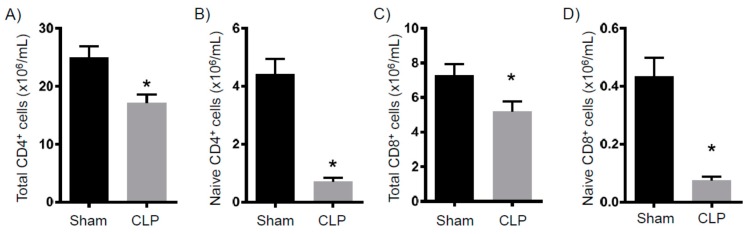
Splenic T cells eight days after CLP. Mice were subjected to either sham surgery (*N* = 16) or CLP (*N* = 16). Eight days later, animals were sacrificed and the spleens were removed. Flow cytometry was then used to characterize the number of (**A**) total CD4^+^ cells, (**B**) naïve CD4^+^ cells, (**C**) total CD8^+^ cells, and (**D**) naïve CD8^+^ cells. * *p* < 0.05 versus sham.

**Figure 4:**
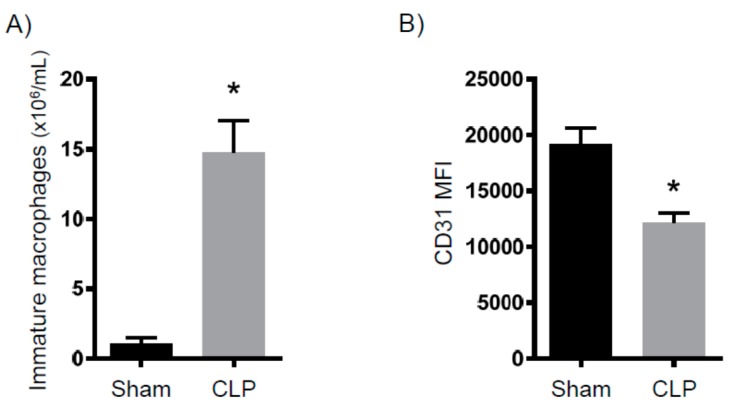
Immature myeloid cells in the spleen eight days after CLP. Mice were subjected to either sham surgery. Eight days later, animals were sacrificed and the spleens were removed. Flow cytometry was then used to characterize the number of (**A**) immature macrophages (sham *N* = 14, CLP *N* = 20) and (**B**) the fluorescent intensity of CD31 as a marker of immaturity (*N* = 8 for both groups). * *p* < 0.05 versus sham.

It is well established that there is significant acute T cell depletion associated with sepsis [8,14]. Further, it has been demonstrated that when this loss is ameliorated, bacterial loads significantly decrease [15]. Thus, the loss of T cells is a key determinant of immune suppression. To determine in our model whether the T cell loss persisted to eight days after CLP, we examined splenic T cell numbers. As shown in Figure 3, the total CD4^+^ and CD8^+^ numbers from septic mice are significantly decreased compared to the controls. Of note, naïve CD4^+^ and CD8^+^ T cell numbers are also dramatically decreased. As immature macrophages have been shown to inhibit T cell proliferation [16], we determined the numbers of these cells and found them to be significantly increased in the CLP mice compared to the control mice (Figure 4A). In addition, CD31, which is a marker of immaturity, is significantly decreased in the CLP mice (Figure 4B). Altogether, we observed a continued decrease in conventional T cells in septic mice, as well as an increase in anti-proliferative macrophages.

### 2.3. Catabolism

**Figure 5:**
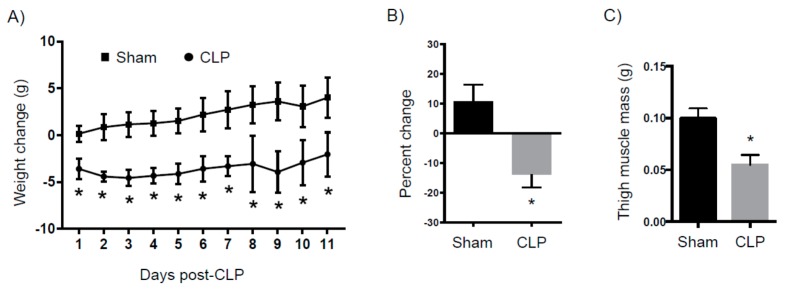
Weight changes after CLP. Mice were subjected to either sham surgery or CLP. Mice were then weighed daily for eleven days. Groups were then compared in terms of (**A**) absolute change in weight and (**B**) maximum percent weight change compared to baseline (*N =* 12 for both groups). (**C**) In another set of experiments, animals were sacrificed after eight days and their thigh muscles were weighed (*N =* 6 for both groups). * *p* < 0.05 versus sham.

Septic patients often display a rapid loss of lean muscle mass due to ongoing catabolism. Here, we examined mice on a daily basis after sham surgery or CLP (Figure 5A). As early as one day after surgery, there was a 5 g difference between the two cohorts that was sustained for the next eleven days. On average, the sham mice gained 10% (95% confidence interval: 1.6 to 19.9%) of their original mass as compared to a loss of 15% (95% confidence interval: −17.1 to −10.4%) in the CLP mice (Figure 5B). Eight days after surgery, CLP mice had a leg muscle mass loss of approximately 50% compared to the sham controls (Figure 5C).

### 2.4. Susceptibility to Infection

**Figure 6:**
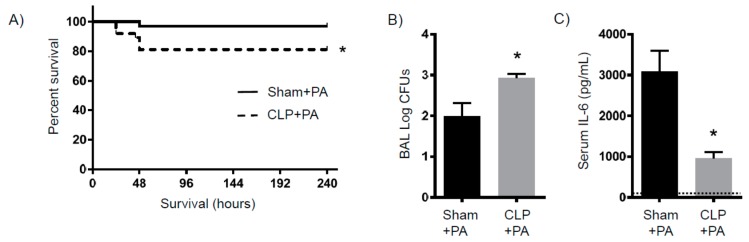
Response to pulmonary infection one week after CLP. Mice were subjected to either sham surgery or CLP. Eight days later, all mice were inoculated with 10^6^ colony forming units (CFU) of *Pseudomonas aeruginosa* (PA) intranasally. Mice were then (**A**) monitored for survival (sham *N* = 32, CLP *N* = 37). In another set of experiments, mice were sacrificed and (**B**) the bronchoalveolar lavage (BAL) was analyzed for CFUs of bacteria 4 h after inoculation (sham *N* = 14, CLP *N =* 20). IL-6 levels in the (**C**) serum were measured at 2 h after inoculation by flow cytometry (sham *N* = 8, CLP *N =* 7). The dashed line indicates serum IL-6 levels in uninfected control mice. * *p* < 0.05 versus sham.

**Figure 7:**
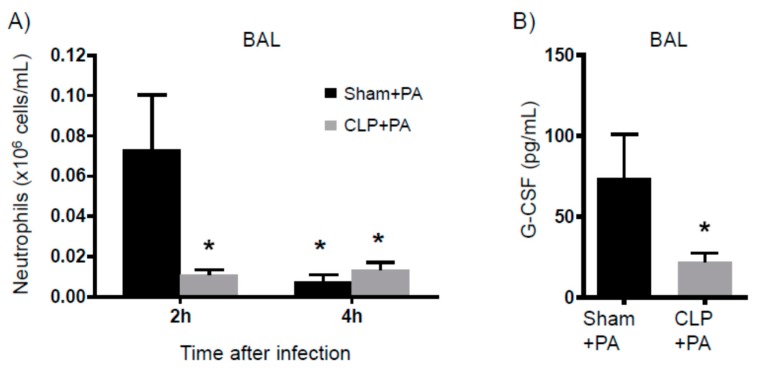
BAL analysis in response to pulmonary infection one week after CLP. Mice were subjected to either sham surgery or CLP. Eight days later, all mice were inoculated with 10^6^ CFU of *Pseudomonas aeruginosa* intranasally. Flow cytometry was then used to evaluate the (**A**) BAL neutrophil numbers 2 hours (sham *N* = 8, CLP *N* = 13) and 4 hours (sham *N =* 14, CLP *N =* 20) after inoculation (**B**) G-CSF levels were then determined 2 h after inoculation when bacterial burdens were the same (sham *N* = 6, CLP *N =* 10). * *p* < 0.05 compared to sham at 2 h. There was no difference between sham + PA and CLP + PA at the 4-h time point.

Altogether, eight days after CLP, the surviving mice had: (A) increased myeloid cells, (B) depleted T cell numbers, and (C) significant body and muscle mass loss. These concurrent characteristics are representative of PICS. An additional characteristic of the PICS population is susceptibility to infection. Therefore, we intranasally inoculated PICS and control mice with *Pseudomonas aeruoginosa* (PA). While the control mice were mostly resistant to the infection, approximately 25% (95% confidence interval: 0.97 to 25.91%) of the PICS mice succumbed to it (Figure 6A). At 2 h after lung infection, the bacterial loads were the same between control-infected and PICS-infected mice (not shown). Four hours after lung infection, however, we found a greater amount of the PA in the lungs of the PICS-infected mice compared to the control-infected mice (95% confidence interval: 1.28 to 2.69 log in the sham versus 2.70 to 3.15 log in the CLP, Figure 6B). The PICS-infected mice exhibited significantly decreased serum IL-6 levels as compared to the control-infected mice at the 2-h time point, despite the bacterial burden being the same (Figure 6C). An analysis of the bronchoalveolar lavage (BAL) fluid revealed a more robust neutrophil accumulation in the control-infected mice within 2 h of infection, which cleared by 4 h, whereas neutrophil accumulation did not occur in the PICS-infected mice at either time point (Figure 7A). In addition, Granulocyte-Colony Stimulating Factor (G-CSF) was significantly lower in the BAL 2 h after infection (Figure 7B). IL-6 and G-CSF levels were not measured at 4 h, since it would be difficult to determine whether any significant differences were related to immune dysfunction or bacterial burden.

**Figure 8:**
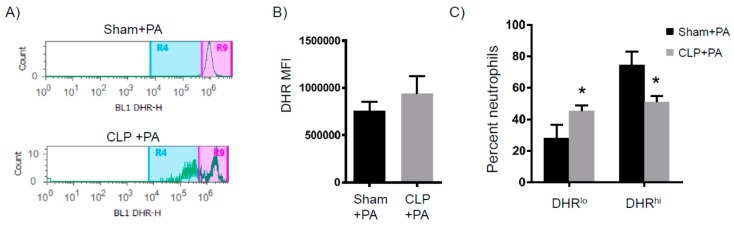
BAL neutrophil oxidative burst in response to pulmonary infection one week after CLP. Mice were subjected to either sham surgery (*N* = 8) or CLP (*N* = 12). Eight days later, all mice were inoculated with 10^6^ CFU of *Pseudomonas aeruginosa* intranasally. Two hours later, BAL cells were analyzed for oxidative burst. (**A**) Representative flow cytometry histograms of the two distinct populations. The BAL neutrophils were then analyzed for (**B**) total DHR levels and (**C**) percent of neutrophils with low versus high DHR. * *p* < 0.05 versus sham.

We next determined the spontaneous reactive oxygen species (ROS) production of BAL neutrophils isolated from either sham-infected or PICS-infected mice at the 2-h time point after infection, when the bacterial burden in the lung was similar. Lung cells from the sham-infected mice had a single group of ROS producing neutrophils, as shown by the single peak of dihydrorhodamine (DHR), which is a fluorescent ROS indicator. In contrast, lung cells from PICS-infected mice had groups of low and high ROS producing neutrophils (Figure 8A). Of note, the intensity from the high ROS in PICS-infected mice was higher than the sham-infected ROS intensity (95% confidence interval: 806,017–952,225 in sham versus 1,674,144–2,301,753 in PICS. In addition, while the total BAL neutrophil ROS from each cohort was not significantly different (Figure 8B), the sham-infected mice had mostly high ROS-producing neutrophils (74%, 95% confidence interval: 55–94%), while the PICS-infected mice had equal numbers of low and high ROS-producing neutrophils in the BAL (Figure 8C. 50% DHR^hi^, 95% confidence interval: 42–59%). Altogether, when given a lung infection, PICS mice had: (1) increased mortality and lung bacterial load, (2) decreased inflammatory response, and (3) dysfunctional neutrophils.

## 3. Discussion

In summary, we have created a PICS model by subjecting mice to a moderate CLP. Mice that survive this insult exhibit ongoing inflammation, immune suppression, and significant weight loss after eight days. Persistent inflammation was marked by significant myelopoiesis in the spleen and bone marrow. This resulted in splenomegaly in which the spleens from PICS mice had a four-fold increase in weight and a nearly five-fold increase in splenocytes. Immune suppression in this model was evidenced by significant depletion in both total and naïve T cells in the PICS mice compared to sham mice, which correlated with a significant increase in the immature macrophage population. These immature macrophages, which have also been called myeloid derived suppressor cells, have been shown to be elevated in human PICS and are thought to contribute to suppressed T cell activity [6,17,18,19]. PICS mice also demonstrated a significant weight loss that was evident within 24 h of injury and extended over eleven days, despite the fact that animals had unlimited access to food and did not have any appreciable changes in activity. In addition, a significant decrease in thigh muscle mass was seen in PICS mice after eight days, indicating that protein breakdown and ongoing catabolism is likely occurring in these mice, similar to humans [20].

When evaluating patients that die from sepsis, most succumb to the illness after a few days or even a few weeks. Death from sepsis is not thought to be related to the infection itself, but from the organ failure that results from the systemic response to the infection. When analyzing these patients post-mortem, about 80% have unresolved septic foci [21]. This implies that, despite the appropriate administration of antibiotics, an inability of the immune system to control the infection leads to ongoing cellular injury and ultimately organ failure. For those that survive the initial insult, the resulting immune suppression renders patients susceptible to secondary nosocomial infections, which significantly increases the risk of late deaths [22]. In our model, about 40% of mice that survived the CLP still had detectable bacterial loads in the peritoneum (data not shown), similar to the observations seen in septic humans. The PICS mice also had a significant susceptibility to a secondary pulmonary infection eight days after the initial insult. Other models of sepsis evaluate this susceptibility to infection during the acute phases of sepsis, when the stress response is still active [23]. Here, we show that the susceptibility to infection persists for many days after the acute stress response. The mechanisms for this sustained immune suppression are unclear. In this model, the decreased bacterial clearance was associated with decreased serum IL-6 and decreased G-CSF levels in the BAL, resulting in decreased neutrophil recruitment to the lung. In addition, two distinct populations of neutrophils were identified in PICS mice: those with a low production of ROS and those with a high production. As shown in many studies, a decreased ability to release ROS is associated with decreased bacterial clearance and excessive ROS production that can lead to tissue destruction. This was different from the sham mice, which only had one distinct group of ROS-producing neutrophils. While this observation is novel and interesting, the total ROS-production of all neutrophils in the lungs of PICS mice was not different from sham mice. Therefore, it is unclear whether the decreased bacterial clearance in the lungs of PICS mice was related to decreased neutrophil recruitment or dysfunctional ROS production from the ones that were present. Overall, this model accurately recapitulates PICS in humans, thus providing a platform to study this process further in mice and identify ways to improve outcomes for these patients. Importantly, each component of PICS occurs simultaneously in this model, which will help determine whether there is a common mechanism to explain this response.

Many have shown that excessive immune cell activation can lead to increased tissue destruction, which is likely responsible for the organ damage that can occur with significant inflammatory insults. On the other hand, others have shown that the depletion of innate or adaptive immune cells leads to significantly decreased bacterial clearance and worse outcomes [24,25]. As a result, many concur that the goal of an effective immune response is to activate the response without an excessive amount of inflammation, achieve source control adequately and efficiently, and then deactivate the response in a timely manner. Since we currently do not know what defines the threshold of “adequate” or “excessive” in each individual, the ability to predict outcomes from a septic insult continues to be difficult. As a result, the ability to predict which patients will develop PICS is challenging. Data from the current study corroborates the notion that a better understanding of the cross talk between the innate and adaptive arms of the immune system is imperative to improving outcomes in PICS [14,23].

Previous results from our lab and others demonstrate that the T cell depletion that occurs in sepsis is related to increased apoptosis [14,26,27]. The current study validates and expands this concept to show that persistent inflammation is associated with prolonged T cell depletion. Efforts to decrease T cell apoptosis, such as the administration of IL-7, significantly improves neutrophil recruitment and increases bacterial clearance without an associated increase in tissue destruction [23,26]. Cyclic AMP (cAMP) is also a potent inhibitor of T cell, neutrophil, and macrophage function [28]. Since cAMP is upregulated in response to many of the stress hormones and cytokines activated during sepsis, further investigation of this pathway in the immune suppression of PICS would be valuable.

Although difficult to determine causality, prolonged catabolism and the loss of lean muscle mass (or sarcopenia) has been implicated in the need for prolonged recovery times for critically ill septic patients [29,30]. This decrease in muscle mass associated with sepsis is not due to a decline in protein synthesis, but rather to an increase in protein breakdown [31]. Many implicate an ongoing inflammatory response as the driving force behind prolonged catabolism in sepsis [32]. Our lab and others are currently studying the pathways involved in this response. However, an interplay between catecholamines, cytokines, and the endocrine response to stress seem to be the driving force behind this process [31,32]. Our PICS model recapitulates the muscle wasting and weight loss seen in septic humans. We did not measure the exact differences in food intake between the sham and CLP groups. However, all animals had unlimited access to food and, in those that survived, the activity and behaviors were similar to sham-injured animals who actually gained weight throughout the course of the study. The exact mechanisms behind this persistent catabolism and the link to the inflammatory response are unclear. This model will be useful to clarify these mechanisms and potentially reveal interventions that can mitigate the profound catabolism that these patients suffer.

The main limitation to this study is the fact that the results from the spleen and bone marrow analyses are difficult to corroborate in humans. However, since peripheral blood monocytes and lymphocytes are easier to obtain, future studies that compare the immune defects in mice with humans suffering from PICS will utilize peripheral blood cells. In addition, we do not have data on the changes in vital signs for the PICS mice. Given that clinical variables are currently used to diagnose sepsis, future studies investigating variables, such as the temperature, heart rate, and blood pressure in mice, will be used to better compare this model to humans. Finally, this model was developed in the absence of surgical debridement or antibiotics to specifically evaluate the immune changes in mice that survive sepsis without variables, such as renal toxicity from antibiotics. This will help determine the mechanisms behind the immune defects in this model in order to accurately develop interventions to prevent progression to chronic critical illness.

In conclusion, we have shown that PICS can be modeled in mice utilizing a moderate CLP method. This model can be used to further evaluate the mechanisms behind persistent inflammation, immune suppression, and catabolism. With an understanding of how PICS develops, we may be able to predict which patients will progress to this state and develop early interventions to mitigate the progression of this syndrome.

## 4. Materials and Methods

### 4.1. Mice

Male CF-1 mice were purchased from the Charles River (Wilmington, MA, USA). For the described studies, six-week-old male mice were obtained and allowed to acclimate for one to two weeks. The mice were housed in standard environmental conditions and were fed a commercial pellet diet and water ad libitum, per the University of Cincinnati Institutional Animal Care and Use Committee (IACUC # 08-09-19-10, 13 November 2014) guidelines.

### 4.2. Cecal Ligation and Puncture

Male mice between seven to eight weeks of age (22–26 g) were used in all experiments. Cecal ligation and puncture (CLP) surgeries were performed as previously described [10,33]. Briefly, CLPs were performed between 8 a.m. and 11 a.m. Mice were anesthetized to effect by 2.5% isoflurane oxygen via a chamber/facemask. The skin was shaved and disinfected with betadine solution. After a 1 cm laparotomy, the latter 33% of the cecum was ligated with a 3-0 silk suture and punctured once on the anti-mesenteric side with a 25-gauge needle, as previously described [12]. Bowel contents were then expressed and wiped via the puncture site to ensure full thickness perforation. Care was taken to ensure that bowel continuity was not obstructed during the procedure. The cecum was replaced into the abdomen and the midline incision was closed in two-layers with a 4-0 silk suture. Animals were resuscitated with 1 mL of sterile saline and were allowed to recover on a 37 °C heating blanket for 60 min. Post-operative analgesics and antibiotics were not used, given that these medications alter or inhibit the immune reactions that we wished to study and reduce the value of these studies [34].

### 4.3. Bacterial Preparation

*Pseudomonas aeruginosa* 762 strain was grown for 16 h on a tryptic soy agar plate (BD Biosciences, Franklin Lakes, NJ, USA) in the 37 °C incubator as previously described [35]. Bacteria was transferred to an Erlenmeyer flask containing 40 mL of tryptic soy broth (BD Biosciences) and was incubated for 60 min at 37 °C with shaking at 125 revolutions per minute. Bacteria was centrifuged at 2800 rpm for 10 min at 21 °C, washed with 40 mL of PBS (Thermo Fischer Scientific; Waltham, MA, USA), centrifuged at the same settings, and then re-suspended in phosphate-buffered saline (PBS). Bacteria was diluted to an optical density of 0.04 using the spectrophotometer based on a standard absorption curve for a desired concentration of 1 × 10^6^ CFUs of bacteria in 20 μL. Eight days after CLP, mice were anesthetized with 3% isoflurane in oxygen. Using a 31-gauge needle on a 1 mL syringe, each mouse was intranasally inoculated with the 20 μL of PA containing the 1 × 10^6^ CFUs of bacteria.

### 4.4. Bronchoalveolar Lavage (BAL)

The trachea was exposed and cannulated with a polyethylene tube connected to a syringe. The lungs were washed by flushing with PBS solution through the tracheal cannula as a 1-mL aliquot and the recovered fluid (approximately 0.75 mL) saved as BAL.

### 4.5. Bacterial Counts

Bacterial counts were performed on aseptically harvested blood by cardiac puncture, as previously described [36]. Samples were serially diluted in sterile saline and cultured on tryptic soy agar plates. Plates were incubated at 37 °C for 24 h and colony counts were performed.

### 4.6. Flow Cytometry

Single cell suspensions were prepared from bone marrow, the spleen, and BAL, as previously described [16,37]. Cell counts were determined using a Coulter AcT 10 cell counter (Beckman Coulter, Brea, CA, USA). Cells were suspended in FACS buffer (PBS with 1% bovine albumin and 0.1% azide) and nonspecific binding to cells was prevented by a pre-incubation with 5% rat serum (Invitrogen, Life Technologies, Grand Island, NY, USA) and 1 μL/sample of Fc Block (BD Pharmingen, San Jose, CA, USA). The following antibodies were all purchased from BD Biosciences (San Jose, CA, USA): Anti-Ly6G (Clone: 1A8), Anti-CD11b (Clone M1/70), Anti-F4/80 (Clone T45-2342), Anti-CD4 (BD Clone RM4-5), anti-TCRβ (Clone H57-597), Anti-CD62L (Clone MEL-14), Anti-CD8 (BD Clone 53-6.7), Anti-Ly-6C (Clone AL-21), and anti-CD31 (Clone MEC 13.3). The following cell types were identified using the following antibody combinations: Neutrophils: Ly-6G and CD11b; Resident macrophages: F4/80 and CD11b; Total CD4 T cells: CD4 and TCRβ (naïve: CD62L^high^); Total CD8 T cells: CD8 and TCRβ (naïve: CD62L^high^); and immature macrophages: Ly-6G^−^, Ly-6C^+^, and CD31^low^. Cells were analyzed using an Attune Acoustic Focusing Cytometer and accompanying software (Life Technologies, Carlsbad, CA, USA), as previously described [38]. The BAL IL-6 and G-CSF levels were determined by a cytometric bead array per the manufacturer’s instructions (BD Biosciences) and as previously described [39]. Spontaneous oxidative burst was determined as previously described [40]. Briefly, a measurement of spontaneous hydrogen peroxide was determined by measuring the oxidation of dihydrorhodamine to fluorescent rhodamine. Cells collected from BAL were first incubated with dihydrorhodamine (DHR; 2.5 × 10^−5^ M) for 5 min at 37 °C. The reaction was stopped by putting cells on ice. Intracellular ROS production was indirectly detected on the FL-1 channel by the rhodamine intensity.

### 4.7. Statistical Analysis

Using Prism 6.0 (GraphPad Software, La Jolla, CA, USA), statistical comparisons were performed with the Mantel-Cox Log-Rank test (survival), Student-test (2 groups), or 1-way ANOVA with the Tukey post hoc comparison (>2 groups). Data were reported as means +/− SEM for experiments containing multiple data points. A *p*-value of ≤ 0.05 was considered statistically significant.
